# Influence of the Inclusion of Chestnut (*Castanea sativa* Miller) in the Finishing Diet and Cooking Technique on the Physicochemical Parameters and Volatile Profile of *Biceps femoris* Muscle

**DOI:** 10.3390/foods9060754

**Published:** 2020-06-06

**Authors:** Noemi Echegaray, Mirian Pateiro, Wangang Zhang, Rubén Domínguez, Paulo C. B. Campagnol, Javier Carballo, José M. Lorenzo

**Affiliations:** 1 Centro Tecnológico de la Carne de Galicia, Rúa Galicia Nº 4, Parque Tecnológico de Galicia, San Cibrao das Viñas, 32900 Ourense, Spain; noemiechegaray@ceteca.net (N.E.); mirianpateiro@ceteca.net (M.P.); rubendominguez@ceteca.net (R.D.); 2 College of Food Science and Technology, Nanjing Agricultural University, Nanjing 210095, China; wangang.zhang@yahoo.com; 3 Departamento de Tecnologia e Ciência de Alimentos, Centro de Ciências Rurais, Universidade Federal de Santa Maria, Santa Maria CEP 97105-900, Rio Grande do Sul, Brazil; paulocampagnol@gmail.com; 4 Área de Tecnología de los Alimentos, Facultad de Ciencias de Ourense, Universidad de Vigo, 32004 Ourense, Spain; carbatec@uvigo.es

**Keywords:** chestnut diet, cooking techniques, colour parameters, cooking loss, shear force, lipid oxidation, volatile compounds, Celta pig breed

## Abstract

The aim of this study was to evaluate the influence of the diet (chestnut vs. commercial feed) and cooking techniques (roasting, grilling, frying and microwaving) on the quality of the *Biceps femoris* muscle of the Celta pig breed. Chemical composition, physicochemical parameters, oxidative stability and volatile profile were analysed. Overall, the inclusion of chestnuts did not affect the chemical composition, except for intramuscular fat content, which was higher in chestnut-fed pigs. The colour and shear force of cooked *Biceps femoris* were not affected by the finishing diet. However, a significant increase in cooking losses and thiobarbituric acid reactive substances (TBARS) value was found with the chestnuts included in the diet. In addition, the inclusion of chestnuts also modified some volatile compound that could be associated with the diet, such as furan, 2-pentyl. On the other hand, the cooking method significantly affected chemical composition (moisture, fat, protein and ash content), colour parameters, cooking loss, TBARS and volatile profile, whereas the shear force was not affected. Concretely, fried and microwave were the techniques that led to a greater presence of intramuscular fat. In addition, the frying method also showed the highest a* value, whereas the microwaved technique displayed the highest cooking loss. Regarding lipid oxidation, the fried method displayed the lower TBARS and hexanal content. On the other hand, the major volatile compounds were aldehydes in all cooking methods except for the frying technique in chestnut samples. Finally, method-frying displayed the lowest amount of total volatiles compounds, unlike grilling.

## 1. Introduction

Nowadays, consumers consider factors such as animal welfare, the high quality of meat and meat derivatives and their traceability. Therefore, the demand for animal products obtained from ancient autochthonous genetic types, fattened in extensive livestock systems has incremented [1]. This is the case of the Celta pig breed, which is a native pig of Galicia (north-west Spain) that is highly valued due to its succulent meat and its intense fat infiltration into lean meat [2]. Furthermore, the Celta pigs are traditionally reared in an extensive or semi-extensive production system using natural resources, such as acorns, chestnut and vegetables. Specifically, one of the most widely used products to fatten this pig is chestnut because it is a great source of carbohydrate [3]. Concretely, these fruits are suitable nourishment for maintaining and rearing adult animals. Moreover, chestnuts are a good source of antioxidants [4] and unsaturated fatty acids [5]. However, chestnut fruits are underused, for instance, the smaller-sized chestnuts and the by-products occasion in the industry. In this area, this livestock feed could be appropriate to obtain high-quality products differentiated by their healthier fat [6] and by their natural endogenous antioxidants. Furthermore, the use of chestnut could also support to reduce the current costs derived from the high prices of commercial concentrates in pig farming [7,8].

On the other hand, the cooking of meat is necessary due to the fact that it becomes edible and more digestible. Besides, cooking causes other positive and negative effects on meat [9] that could influence consumer acceptance. In view of these developments, it is important to understand the events that take place during the heat treatment of meat. Thus, lipid oxidation and Maillard reactions take special interest because they are important mechanisms for the development of odour, flavour and colour in cooked meat [10]. Regarding nutritional value, cooking changes the chemical composition of meat [11]. This process may cause nutritional losses such as thermolabile vitamins, and it may produce several toxic compounds like heterocyclic amines and cholesterol oxidation products (COPs) [9]. In terms of physicochemical parameters, meat colour is principally affected by myoglobin, which can be found in three different states (deoxymyoglobin, metmyoglobin and oxymyoglobin) and in distinct concentrations, depending on the heat treatment applied [12]. Also both water holding capacity and texture profile can be affected due to modifications in the structure of proteins, mainly myofibrillar [13] and the connective tissues due to changes caused by cooking. Moreover, other chemical reactions could also influence the appearance, flavour and nutrient contents of final cooked meat, transforming its quality and its consumer’s acceptability [13]. In this regard, the profile of volatile compounds is principally affected by the lipid oxidation routes, since these generate a large amount of desirable aromatic compounds, which are the most important source for taste and odour formation [9]. Additionally, the products derived from the Maillard’s reaction are also important in the meat flavour [14].

All the reactions explained above occur differently depending on the cooking method used; that is to say, they depend on the temperature reached by meat sample and on the time employed for the treatment [15]. Thus, different common cooking methods such as roasting, grilling, frying, and microwaving will have different effects on the nutritional, physicochemical parameters and volatile profile of the cooked meat. On this matter, several studies have been accomplished. Broncano et al. [9] observed that grilled pig steaks were the least affected by lipid oxidation with respect to roasted, frying and microwaved pig fillets. In addition, Domínguez et al. [15] displayed that cooking losses were significantly affected by heat treatment in foal meat, being higher after microwaving and lower after grilling. At the same time, thiobarbituric acid reactive substances (TBARS) value was significantly higher when foal steaks were roasted or microwaved. A similar trend was observed by Lorenzo and Domínguez [14] in the same type of meat. Besides, Domínguez et al. [11] determined that frying provided a lower content of saturated fatty acids, due to the incorporation of monounsaturated fatty acids from frying oil compared to other thermal treatments (roasted, grilled and microwaved). Finally, several works determined that the generation of volatile compounds also seemed to be directly related to cooking treatment [11,14,16].

Therefore, the aim of the current work was to know the effect of both, the inclusion of chestnuts (*Castanea sativa* Mill.) in the finishing diet of Celta pigs and the use of four common cooking techniques (roasted, grilled, fried and microwaved) on chemical composition, colour parameters, cooking loss, lipid oxidation, shear force and volatile compounds of pork *Biceps femoris* slices.

## 2. Materials and Methods

### 2.1. Experiment Design and Slaughtered

For this research, 18 Celta pigs (10 males and 8 females) were raised in a semi-extensive system. The piglets were vaccinated and deparasitised according to the standard protocols and they were weaned at 40 days. After breastfeeding, the pigs were fed with a commercial compound in a semi-extensive regime with a livestock density of 12 animals per hectare. Piglets of both sexes were castrated at the age of 2 months for males and 3 months for females, according to the Council Directive 2008/120/EC [17]. At the age of 8 months, the hogs were randomly divided into 2 distinct groups of 9 animals (5 males and 4 females). They were guarded in different localizations of land for their distinct feeding during the finishing period (4 months), warranting that there was not any other form of nourishment that the pigs had access to. One of the groups after receiving a temporary combination diet (commercial compound feed/chestnuts; 1.5 kg commercial compound/3 kg chestnuts animal^−1^ d^−1^) during the ninth month, were supplied only with chestnuts (6 kg per head and day) in the remaining three months previous to slaughter (CH group). The pigs of the other group were fed with 3 kg of commercial compound feed per head and day, until the slaughter age (CF group). All animals were slaughtered at 12 months age, with a mean live weight of 149 107.53 ± 8.26 and 115.41 ± 9.15 kg; *P* > 0.05 for chestnut and commercial feed diets, respectively. The chemical and fatty acid composition of both diets supplied (chestnuts and commercial compound feed) were those indicated in a previous study [18].

At the end of the fattening, pigs were carried 80 km to a commercial abattoir (Frigolouro, Porriño, Pontevedra, Spain) and were kept for 12 h without access to food, although they did have access to water. Pigs were slaughtered by electrical stunning and exsanguination. Next, they were scalded, skinned and eviscerated in accordance with standard commercial procedures. Straight away, carcasses were chilled at 4 ± 1 °C in a cold chamber during 24 h. After the refrigeration term, samples from the *Biceps femoris* muscle from each carcass were removed, vacuum packed and warehoused at 4 ± 1 °C down to processing the next day.

### 2.2. Sample Preparation and Cooking Process

From each animal, the *Biceps femoris* muscle were cut into 16 slices (1.5 cm thick) and divided into 4 groups according to the following cooking methods: roasted at 200 °C for 10 min, utilizing an electrical oven (Rational SCC101, Barcelona, Spain); grilled at 130–150 °C during 5 min on each surface, employing an electrical griddle (Delonghi CG660, Treviso, Italy); fried with 15 ml of refined olive oil, at 170–180 °C during 5 min on each surface; and microwaved at 1000 W for 1.5 min on each surface in a microwave oven (Panasonic, NE-1037, Osaka, Japan). The cooking treatment was considered finished when all the samples reached an internal temperature of 75 ± 2 °C. A total of 288 samples (4 slices per animal and cooking method × 9 animals × 2 feeding groups × 4 cooking methods) were cooked and the cooking parameters (temperatures and times) were monitored throughout the process. After cooking, the slices were cooled at room temperature (25 °C) and colour parameters, cooking loss, and shear force were determined. Then, the samples were ground and lipid oxidation was analysed. In addition, the excess sample was vacuum packed and stored at −80 °C for no longer than four weeks until chemical composition and volatile compounds analysis were completed.

### 2.3. Analysis of Proximate Composition

Moisture [19], protein (Kjeldahl *N* × 6.25) [20] and ash [21] were quantified and expressed as percentage according to the ISO recommended standards, while fat was determined following the American Oil Chemistry Society (AOCS) official procedure [22]. In brief, moisture value was calculated through the weight loss tested by 5 g of sample maintained in an oven (Memmert UFP 600, Schwabach, Germany) at 105 °C, until constant weight. For the analysis of intramuscular fat content, 1 g of samples were subjected to a liquid–solid extraction employing petroleum ether during 60 min, in an extractor apparatus (Ankom^HCI^ Hydrolysis System XT10, Macedon, NY, USA). Intramuscular fat percentage was obtained by gravimetric difference. Protein content was calculated according to Kjeldahl Total Nitrogen method, multiplying the total nitrogen content by 6.25. For this, 1 g of samples was subjected to reaction with sulphuric acid (using cuprum sulphate as a catalyst) in a digester (Gerhardt Kjeldahl KB20 Vapodest^®^, Königswinter, Germany). Organic nitrogen was transformed to ammonium sulphate, which was distilled in alkali conditions in a distillation apparatus (Gerhardt^®^ Vapodest 50 Carrousel, Königswinter, Germany). The ammonium freed was captured in a boric acid solution where it was titrated using a hydrochloric acid solution. Finally, ash content was determined by calcining 3 g of sample at 600 °C in a muffle furnace (Carbolite^®^ RWF 12-13, Hope Valley, England) into a porcelain capsule down to constant weight.

### 2.4. Colour Analysis

Colour parameters were measured on the surface of the cooked pork muscle utilizing a portable CR-400 colorimeter (Konica Minolta Sensing Inc., Osaka, Japan). The outcomes were expressed in the CIELAB space as lightness (L*), redness (a*), and yellowness (b*). Colour was determined at three different points in every slice.

### 2.5. Cooking Loss

After thermal treatment, the samples were cooled for 30 min at room temperature and the percentage of cooking loss was obtained by determining the difference in weight among the cooked and raw samples, as follows:

(1) $$\%\;Cooking\;loss=\frac{Raw\;meat\;weight-Cooked\;meat\;weight\;}{Raw\;meat\;weight}\;\times \;100$$

### 2.6. Texture Measurement

The Warner–Bratzler (WB) test was used to determine the texture profile employed a texture analyser (TA-XT2, Stable MicroSystems, Godalming, UK). Three cooked meat cuts of 1 × 1 × 2.5 cm (height × width × length) were received parallel to the muscle fibre direction. Next, the pieces were utterly cut perpendicular to the muscle fibre direction at a crosshead speed of 3.33 mm s^−1^ using a WB shear blade with a triangular slot cutting edge (1 mm of thickness). Maximum shear force was obtained and shown by the highest peak of the force-time curve. This factor exemplified the maximum resistance of the sample to the cut.

### 2.7. Lipid Oxidation

Lipid stability was assessed using the 2-thiobarbituric acid (TBA) method suggested by Vyncke [23]. An aliquot of 2 g of homogenized cooked sample was scattered in 10 ml 5% trichloroacetic acid for 2 min, using an Ultra-Turrax (Ika T25 basic, Staufen, Germany). The homogenate was maintained at −10 °C during 10 min and centrifuged at 3500 rpm for 10 min. The supernatant was filtered through a Whatman Nº 1 filter paper. Then, 5 ml of filtrate was reacted with 5 ml of 0.02 M TBA solution and incubated in a water bath at 97 °C during 40 min. Finally, the samples were cooled at room temperature and the absorbance was determined at 532 nm. A standard curve of malonaldehyde with 1,1-3,3 tetraetoxipropane (TEP) was used to obtain the thiobarbituric acid reactive substances (TBARS) value, which was expressed as mg malonaldehyde concentration (MDA) per kg of muscle.

### 2.8. Volatile Compound Profile

A gas chromatographic 6890N (Agilent Technologies, Santa Clara, CA, USA) fitted with a DB-624 capillary column (30 m, 0.25 mm i.d., 1.4 μm film thickness; J&W Scientific, Folsom, CA, USA) coupled to a mass selective detector 5973N (Agilent Technologies) was used to determine the volatile profile. For this, the volatile compounds were extracted using a solid-phase microextraction (SPME) device (Supelco, Bellefonte, PA, USA), including a fused silica fibre (10 mm length) coated with a 50/30 μm thickness layer of divinylbenzene/carboxen/polydimethylsiloxane (DVB/CAR/PDMS) and following the method explained by Domínguez et al. [16] with some modifications. Initially, 1 g of minced sample was weighed into a 24-ml glass vial. This vial was screw-capped with a laminated Teflon-rubber disc. Then, it was maintained during 15 min in an oven at 35 °C to equilibrate the volatile compounds in the headspace, guarantying a homogeneous temperature for both sample and headspace. Afterwards, the fibre (previously prepared by heating in a gas chromatograph injection port at 270 °C during 60 min) was inserted into the glass vial across the septum and exhibited to headspace for 30 min, at 35 °C in the oven. Once removal was complete, the fibre was transferred to the injection port of the gas chromatograph–mass spectrometer (GC–MS) system. The SPME fibre was desorbed and kept in the nozzle port at 260 °C for 8 min in splitless mode. Helium was employed with a linear velocity of 40 cm s^−1^ as a carrier gas. The temperature programme was initially isothermal at 40 ºC during 10 min, then lifted to 200 °C at 5 °C min^−1^ and after to 250 °C at 20 °C min^−1^, and lastly maintained for 5 min (the total run time was 49.5 min). Injector and detector temperatures were both supported at 260 °C.

The mass spectra were achieved employing a mass selective detector working in electronic impact at 70 eV, with a multiplier voltage of 1953 V and collecting data at 6.34 scans s^−1^ over the range *m*/*z* 40–300. Volatile compounds were identified by comparing their mass spectra with those shown in the NIST05 (National Institute of Standards and Technology, Gaithersburg) library (>80% of coincidence), and/or by comparing their mass spectra and retention time with authentic standards (Supelco, Bellefonte, PA, USA), and/or by the calculation of the retention index relative to a series of standard alkanes (C_5_-C_14_) (for calculating Kovats indexes, Supelco 44585-U, Bellefonte, PA, USA) and matching them with data relayed in literature. The aftermaths obtained are showed as area units (AU) × 10^6^ per g of dry matter.

### 2.9. Statistical Analysis

A total of 288 samples (4 slices per animal and cooking treatment × 9 animals per finishing diet × 2 finishing diets × 4 cooking treatments) were analysed in the present research. Normal distribution and homogeneity of variance were previously tested (Shapiro–Wilk). With the objective of determining the sway of feeding and of different cooking methods on the parameters investigated, an analysis of variance (ANOVA) using the General Linear Model (GLM) procedure of the SPSS package version 23.0 (IBM SPSS, Chicago, IL, USA) was accomplish. Analysis of every parameter and the significance was given as *P* < 0.05, *P* < 0.01 and *P* < 0.001. Subsequently, Duncan’s test with a 0.05 level of significance was carried out. Moreover, the Pearson’s linear coefficient was used to determine correlations between variables implemented with the same SPSS package.

## 3. Results and Discussion

### 3.1. Chemical Composition

In relation to the chemical composition of the *Biceps femoris* muscle, the results are shown as a percentage in Table 1. As we can see, the diet significantly (*P* < 0.05) affected the fat content in all thermal treatments. Moreover, CF pigs presented a higher (*P* < 0.05) amount of ash in roasted samples and a higher (*P* < 0.05) content of moisture in fried and microwaved samples than in CH. Regarding the fat and moisture content, the results reported in our research are in accordance with those stated by Pugliese et al. [24], who determined that an increase in the ration of chestnut in the pig’s diet favoured the accumulation of intramuscular fat to the detriment of moisture content. The observed increase in intramuscular fat could be due to the higher energy/protein ratio of chestnuts (65.6; data not shown) in comparison with this ratio in compound feed (29.8; data not shown), which promotes an increase in fat deposition during fattening of pigs [24]. However, other studies showed that the inclusion of chestnuts did not significantly affect the muscle composition of pigs, although it did always increase the fat content in a non-significant way [7]. With respect to the protein percentage, it was not significantly affected by the finishing diet. Meanwhile, for the ash content, a higher value (*P* < 0.05) was obtained in CF pigs only in roasted samples, while the other samples did not show differences. Generally speaking, the inclusion of the chestnut significantly increases the fat content, while its influence on the other parameters was not as clear, depending on the cooking method. Thus, a limited influence of diet in the chemical composition of the *Biceps femoris* muscle was observed in the present research. These finding agree with those reported by Temperan et al. [7], who reported that the chemical composition of the *Longissimus dorsi* and *Semimembranosus* muscles of Celta pigs was not influenced by the inclusion of chestnut in the pig diet.

On the other hand, as a result of the different cooking methods, chemical composition parameters were significantly (*P* < 0.05) affected. In CH pigs, moisture (*P* < 0.01), protein (*P* < 0.01) and ash (*P* < 0.05) amounts were affected by the cooking treatment, while in the CF pigs, the contents of fat (*P* < 0.01), protein (*P* < 0.05) and ash (*P* < 0.05) varied among the treatments. As occurs in CH samples, Broncano et al. [9] also found that the different cooking methods did not significantly affect the intramuscular fat content. However, as commented above, the fat percentage of slices from CF pigs was affected by heat treatment. This fact, although disagreeing with that obtained with Broncano et al. [9], coincides with the results achieved by Serrano et al. [25], who also determined the existence of significant differences in the concentration of fat after applying different cooking methods. More specifically, in our case, grilled and roasted treatments showed the lowest fat in CF pig samples (2.67%). Meanwhile, the fried method obtained the highest fat values (4.07% for CF pig muscle). The increase in fat undergone during frying may occur as a consequence of the incorporation of lipids from the frying oil itself used into the meat, as seen in previous works [25]. The trend in the percentage of moisture was the same for diets, displaying the lowest values in the microwave cooking (53.41 and 58.96% for CH and CF, respectively) and the highest percentages in the grilled treatment (60.89% for CH and 62.46% for CF). This fact is due to microwaved samples presenting the highest cooking losses, while grilled showed the lowest cooking loss values. The same behaviour was reported by other authors, who found in foal meat that the samples treated with a microwave had significantly higher cooking loss values [14,15,16]. The same authors also reported that grilled samples had the lowest cooking losses, while the other treatments (fried and roasted) presented intermediate values. Nevertheless, the meat with the highest moisture content obtained the lowest percentages for the protein and ash. In this way, the grilled samples displayed a percentage of protein of 32.06 and 32.40% for CH and CF, respectively, and an ash content value of 1.59% for CH and of 1.61% for CF samples. On its behalf, microwaved samples, which showed a lower moisture value, displayed the highest content of protein from CF slices (35.99%) and the second highest percentage in case of CF slices (36.07%). This fact could be due to the effect of the concentration of the compounds because of water loss. Finally, the ash content was higher in fried samples (1.79%) for CH pigs and roasted (1.83%) for CF pigs.

### 3.2. Colour Parameters

The results corresponding to the colour parameters of pig *Biceps femoris* slices cooked using different methods are reported in Table 2. The outcomes obtained for the values of L* (34.89–52.32), a* (5.89–10.32) and b* (15.81–19.63) were similar to those found by other studies. In this way, several authors reported data between 48 and 81 for L*, between 0.3 and 11 for a*, and among 10 and 24 for b* for cooked pork muscle and derived products [13,26,27,28,29]. As we can observe, all the colour parameters of our work are in the range of these authors, except for the L* value, which was a little lower in the case of frying for both diets (34.89 for CH and 40.69 for CF diet). In the present study, the diet has not significantly affected the colour parameters, except in the fried samples, where the value of b* was altered, being higher in slices of CF pigs (19.63) than in CH samples (15.81). These results agree with those reported by Temperan et al. [7], who also did not find significant differences in the meat colour of Celta pigs fed with commercial feed or with two levels of chestnut.

Then again, the different cooking methods did significantly (*P* < 0.01) affect all the parameters in both diets, with the exception of yellowness of CH pigs. The significant differences derived from the distinct heat treatments could be due to the interconversion of myoglobin in deoxymyoglobin, metmyoglobin and oxymyoglobin. This interconversion takes place through different reactions of oxygenation, oxidation and reduction, which influences the external appearance of meat colour [30]. Aforementioned interconversions and degradations can occur in different ways, depending on the cooking time and temperature used. Thus, different degrees of denaturation of the red heme proteins are generated according to the culinary method used [31]. For instance, Bertola et al. [32] observed that the temperature caused a denaturation of myoglobin and haemoglobin which generated brown precipitates in the meat. Comparing our results with a previous study carried out on raw *Biceps femoris* from Celta pig [33] (L* 48.9; a* 18.1; b* 13.1), a decrease in luminosity (L*) was observed in the case of fried samples (34.89 for CH and 40.96 for CF slices), while, for the rest of the cooking treatments, there seemed to be an increase in this parameter with respect to raw *Biceps femoris* muscle (L* among 49.25–52.32). In particular, the highest value of L* was obtained for microwaved (51.22 and 51.33 for CH and CF slices, respectively) and for grilled (51.13 for CH and 52.32 for CF slices).

In relation to the value of redness (a*), there was a decrease in this parameter in all culinary treatments with respect to uncooked *Biceps femoris* muscle, where an a* value of 18.1 was found [33]. More specifically, frying was the method that provided the highest value for both diets (9.34 for CH and 10.32 for CF slices) followed by roasted (7.15 and 7.52 for CH and CF slices, respectively), grilled (6.31 for CH and 6.39 for CF samples) and microwaved (5.89 and 6.34 for CH and CF samples, respectively). In general terms, these facts agree with those obtained by others who observed that cooking increased the L* value while generating a loss of redness (a*) in foal [34] and beef meat [35]. More concretely, Lorenzo et al. [34] observed a similar trend to our study, since they reported a lower L* value for fried foal meat with respect to roasted, grilled or microwaved samples. Simultaneously, Lorenzo et al. [34] obtained the highest a* values for fried foal meat, while the lowest were for microwaved meat, as in our work. Finally, the value of yellowness (b*) increased with the cooking treatments (15.8–19.6), in comparison with the raw *Biceps femoris* muscle (13.1) [33]. In this case, only the CH pigs showed significant differences among cooking treatments. The highest values were obtained for roasted (18.95) and grilled (17.54), being the lowest for frying (15.81).

### 3.3. Cooking Loss

The different meat cooking treatments lead to a reduction in weight mainly owing to leaks in the water content of meat. However, water is not the only compound lost during cooking, but other water-soluble components also escaped [16]. This loss of mass occurs principally due to changes in protein chains as a consequence of increased temperature. Specifically, after an overtake of 40 °C, myofibrillar proteins shrink, favouring coagulation and strengthening the structures of the muscle fibre. All that involves a reduction in the water retention capacity [27]. In this way, cooking losses are dependent on the temperature, on the rate of heating and on the mass transfer process. Hence, the use of different cooking methods will demarcate the differences in water holding capacity of meat, therefore also affecting sensory qualities as tenderness and juiciness [36].

The values determined for cooking losses of pig slices treated by different methods (Table 2) were comparable to those reported in distinct pig muscles by other authors who obtained values ranging between 10–43% [24,37,38]. With respect to the use of chestnut in the finishing diet, this seemed to significantly (*P* < 0.05) increase the cooking loss in all heat treatments, except in grilled. These aftermaths are in disagreement with those obtained by Temperan et al. [7] who did not observe that the inclusion of chestnut affected the cooking loss. Nonetheless, our results agree with those showed by Pugliese et al. [24] who also found that the use of chestnut in the pig diet significantly affected this parameter. These differences could be caused by the existence of non-aqueous fluid losses in addition to water leak, since high temperatures can melt the fat and degrade the structures that contain it [24]. In this respect, it would be logical to think that the initial fat content of raw meat belonging to CH pigs was higher, since in cooked samples this content was significantly (*P* < 0.05) higher than commercial feed slices (Table 1). This upper content could favour greater losses of non-aqueous fluid during the culinary treatment, therefore increasing cooking losses in pigs fed with chestnut [24]. On the other hand, cooking treatment also significantly (*P* < 0.05) affected cooking losses (Table 2). The same trend was observed in both diets. The microwaved slices showed the highest cooking loss values (45.43 and 40.66% for CH and CF pigs, respectively), followed by fried samples (42.93% for CH and 37.55% for CF samples) and roasted slices (39.34 and 35.02% for CH and CF pigs, respectively). On the contrary, the grilled samples showed the lowest cooking losses (35.90 and 33.46% for CH and CF samples, respectively). Nevertheless, the cooking losses found by us were similar to those observed by several other authors [14,25,34] who determined that microwaving increased the losses in different meats of beef, foal and chicken. The differences in cooking losses found in this study could be due to the appearance of an external layer in samples treated by roasting, grilling and frying, while, in the microwave process, this protective rind is not generated [25,39]. Besides, the structure and function of protein may also be influenced as a result of the high electromagnetic field used by microwaved, because this generates aggregates that can favour the release of water [40]. Moreover, grilled samples presented the lowest cooking losses and they were similar to those obtained for roasted. As we said, these facts respond to the appearance of an external crust on the surface of the cooked samples, which could act as a physical barrier preventing liquid losses [13,31]. Furthermore, roasting increases the viscosity of the cooking liquid by favouring the dissolving of the intracellular material in this liquid, thus making its loss more difficult [14]. Oppositely, according to Juárez et al. [41], frying leads to enough high cooking losses (42.93% for chestnut and 37.55% for commercial feed) even generating a protective crust. These aftermaths could be due to a high temperature being used in this method (170–180 °C) compared with the grilled temperature (130–150 °C), which causes an increase in myoglobin degradation [35]. Furthermore, in our work, a negative and significant correlation among the percentages of cooking loss and moisture was observed in meat both from CH pigs (r = −0.621; *P* < 0.01) and CF pigs (r = −0.818; *P* < 0.01). In this way, it is confirmed that although there are other liquids that contribute to cooking losses, the water is closely correlated with this parameter.

### 3.4. Texture Analysis

Texture is one of the most important sensory qualities of meat, affecting consumer acceptance [27]. Inside the texture parameters, shear force is a good indicator of meat hardness. These criteria supply good information about the degree of denaturation of myofibrillar proteins (fundamentally of the actomyosin complex), as a consequence of the contraction of muscle fibres produced by the cooking treatment [42]. Then again, collagen is also a compound that has been related to the hardness of meat, but previous studies have shown that, in the case of pork meat, it showed low correlation coefficients with tenderness [43]. The outcomes obtained for the shear force in our research (Table 2) were slightly higher than those obtained by other researchers [44,45,46] in the *Longissimus* pig muscle, since the shear force determined by them was between 32 and 49 N. Moreover, the values found in the present research were also higher than those described in the raw *Biceps femoris* muscle (about 18 N) from Celta pig [33]. This fact was expected, since raw meat is not comparable to cooked meat. In contrast, Pugliese et al. [24] and Temperan et al. [7] obtained higher values than our in *Longissimus* pig muscle (100–135 N). These differences in texture may be due to the fact that Celta pigs, like those used in this study, present greater physical activity during breeding and their slaughter age is higher compared to pigs of improved breeds [7]. Additionally, as reported by Temperan et al. [7], the type of muscle affected the texture parameters. Thus, the different muscles studied among researches could also explain the differences. Following hardness, all of our slices could be considered as “though” according to Destefanis et al. [47], since the values obtained for this parameter were higher than 52.68 N. In the present study, the shear force value was not influenced by diet of cooking treatment. This fact is in accordance with data obtained by Pugliese et al. [24] and Temperan et al. [7], who observed that the inclusion of chestnut in pigs diet did not significantly affect texture parameters of meat. Regarding cooking treatments, several works showed that tenderness was strongly influenced by the temperature and cooking time [13,48]. Even so, with the outcomes achieved, it seems that there is a tendency for the fried treatment to increase the hardness of the samples from CH pigs (81.18 N) with respect to the other ones (77.09, 73.95 and 73.50 N for microwaved, roasted and grilled treatments, respectively). In the case of CF pigs, roasted samples obtained the highest shear force (86.92 N), followed by fried (80.90 N) and microwaved (77.57 N) samples. Eventually, grilled was the method that supplied the least hardness for both diets (73.50 N for CH and 71.43 N for CF samples). In the same line, Lorenzo et al. [34] evidenced this trend, because they concluded that grilling in foal meat also generated less hardness slices compared to other treatments (roasted, fried and microwaved). Besides, according to Ismail et al. [49] the Pearson correlation demonstrated that shear force was positively related to cooking loss (r = 0.301; *P* > 0.05; and r = 0.562; *P* < 0.01 for CH and CF, respectively). However, there were only significant (*P* < 0.01) differences in CF samples.

### 3.5. TBARS Values

**Figure 1:**
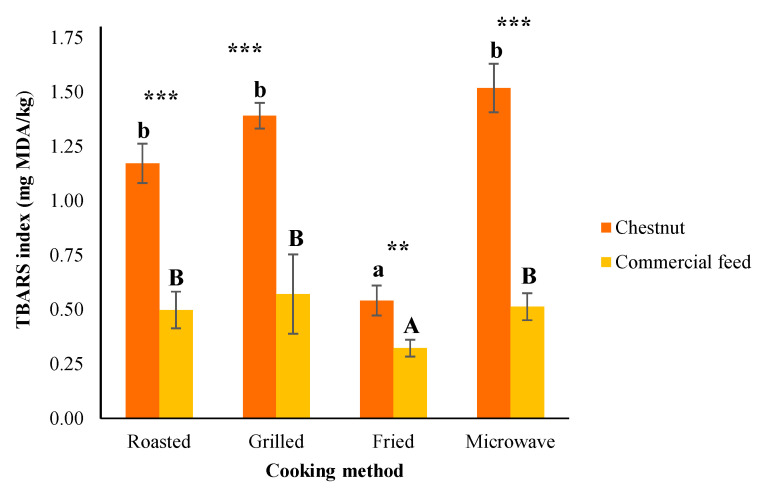
Effects of the inclusion of chestnut in the finishing diet and of cooking methods on the thiobarbituric acid reactive substances (TBARS) index of Celta pig *Biceps femoris* (mean ± standard error). Influenced by feeding: * (*P* < 0.05); ** (*P* < 0.01); *** (*P* < 0.001); ns: no significant difference. Different letters within the same feeding regimen (chestnut (^a,b^) or commercial feed (^A,B^)) indicate significant differences for cooking methods (*P* < 0.05).

During cooking, a moderate lipid oxidation occurs, which is an important source for the formation of meat flavour and odour compounds [9,50]. In this research, this oxidation was determined using the TBARS value, meaning an index of malonaldehyde concentration (MDA), which is a secondary product of lipid oxidation [51]. The results, expressed as mg MDA/kg of the *Biceps femoris* muscle, are presented in Figure 1. As we can see, there were significant (*P* < 0.01) differences regarding the diet provided in all thermal treatments. More concretely, the inclusion of chestnuts in the finishing diet seemed to increase the value of TBARS. These outcomes do not agree with those shown previously by Cobos et al. [52] and Díaz et al. [53], who observed that the inclusion of chestnuts reduced the TBARS index in pork meat and its derivates. At the same time, our research also do not agree with other studies that did not find any significant difference in lipid oxidation after using chestnuts in pig fattening [54,55,56,57]. The increase in TBARS value that occurred in our inquiry could be due to the fact that CH samples present a higher percentage of intramuscular fat (Table 1); therefore, it would be normal to present a higher oxidation. However, in our research, it is difficult to explain this because we have seen that TBARS are not correlated with the percentage of fat, since very low Pearson correlations were obtained for both diets (r = 0.047; *P* < 0.05; and r = −0.073; *P* > 0.05, for CH and CF, respectively). Even so, a possible reason for the datum obtained could be that CH pigs tend to accumulate a higher concentration of unsaturated fatty acids [6,8,18], which are more susceptible to oxidation [51,58,59].

In relation to the different thermal treatments, we have obtained TBARS values ranging from 0.32 to 1.52 mg MDA/kg. These aftermaths are similar to those reporter in different cooked pig meat and products where values between 0.29 and 3.26 mg MDA/kg were come across [9,27,60,61]. However, other authors found lower TBARS rates, which did not exceed 0.29 mg MDA/kg [62,63,64]. One reason why our study shows high values of TBARS may be the fact that the Celta pig breed tends to accumulate more unsaturated fatty acids than other breeds [2]. Additionally, Tarladgis et al. [65] indicated that the TBARS value from which the perception of rancidity dominates the flavour of the pig cooked meat was between 0.5–1 mg MDA/kg. According to the previous rank, the samples belonging to the pigs fed with chestnut would present a rancid flavour, since they obtained a TBARS index higher than 1 mg MDA/kg (except for frying method). However, other studies for distinct products showed less restrictive values. Thus, Greene and Cumuze [66] and Campo et al. [67] showed a threshold value over 2 mg MDA/kg for beef meat.

On the other hand, the different cooking methods significantly (*P* < 0.01) affected the TBARS index for both diets. Fried samples presented significantly lower values (*P* < 0.05) of TBARS than the other cooking methods that did not differ significantly among them. More concretely, microwaved and grilled treatments displayed the highest TBARS values (1.52 and 1.39 mg MDA/kg for CH samples and 0.51 and 0.57 mg MDA/kg in CF samples, respectively). These outcomes do not agree with those obtained by Soladoye et al. [68], who observed that microwave cooking did not generate large TBARS values in bacon. But other authors obtained results that adapt to ours. Thus, Hernández et al. [60], Weber et al. [69] and Lorenzo et al. [34] concluded that the microwaving produced a greater lipid oxidation than other thermal treatments. Keep in mind that this cooking method is characterized by the use of a low time and temperature, the data obtained in this work suggest that there may be an interaction between microwave and meat fat which causes the oxidation of unsaturated fatty acids [9]. On the contrary, as commented above, frying was the culinary method that obtained the lowest value of TBARS for both diets (0.54 ad 0.32 mg MDA/kg for CH and CF samples, respectively). This result agrees with those reported by Weber et al. [69] and Lorenzo and Domínguez [14]. This may be due to the decomposition and volatilization of the secondary oxidation compounds generated by the high treatment temperature [60,70]. At the same time, lipid oxidation products could be lost by dissolving in the frying oil [69]. Some authors even reported the possibility that the high polyphenol content in the oil could act against oxidation [71]. However, other works displayed that frying with vegetable oils generated greater oxidation, which could be due to the oxidation of polyunsaturated fatty acids coming from the oil [9,25]. Additionally, roasted treatment showed intermediate values for the TBARS index (1.17 mg MDA/kg for CH samples, and 0.57 kg MDA/kg for CF samples). Lastly, it should be noted that any studies observed that lipid and pigment oxidation are closely interrelated [72]. This is the case of our investigation, where a negative Pearson correlation is observed between the TBARS and a* value of chestnut and commercial feed slices (r = −0.770; *P* < 0.01, and r = −0.548; *P* < 0.001 respectively), being significantly different for both diets.

### 3.6. Volatile Compounds

The cooking of meat releases various volatile compounds through different reaction mechanisms. These procedures are primarily due to the Maillard reaction, lipid degradation and oxidation, as well as the interaction among their intermediary compounds [73]. In our research, a whole of 52 volatile compounds from *Biceps femoris* muscle were identified in the head space of the cooked samples using the SPME/GC-MS method. Table 3 shows the total compounds of each chemical family and Table 4 displays the individual compounds handed out into five chemical families (aliphatic hydrocarbons, aromatic and alicyclic hydrocarbons, alcohols, aldehydes and “others”).

According to the diet provided, the families of aliphatic hydrocarbons and aromatic and alicyclic hydrocarbons were significantly (*P* < 0.01) affected in all the cooking methods. The groups of aldehydes and “others” were also affected (*P* < 0.05) significantly in all culinary treatments, except in the grilled cooking. However, the alcohols group were hardly altered by diet, as only significant (*P* < 0.05) differences were observed in roasted slices (Table 3). Additionally, the inclusion of chestnut in the pig diet significantly (*P* < 0.05) affected the levels of 28, 18, 29 and 27 volatile compounds in roasted, grilled, fried and microwaved samples, respectively (Table 4). Regarding the cooking method, significant (*P* < 0.001) differences were found in all five family groups for both feeds, except for the alcohol group in CH samples (Table 3). More concretely, the heat treatment had a significant (*P* < 0.05) impact on all volatile compounds quantified, apart from 2,2,4,4-tetramethyloctane, octane and 2,3-butanediol in CH slices and decane, octane and pentane, 2,3,4-trimethyl- in CF samples (Table 4).

**Figure 2:**
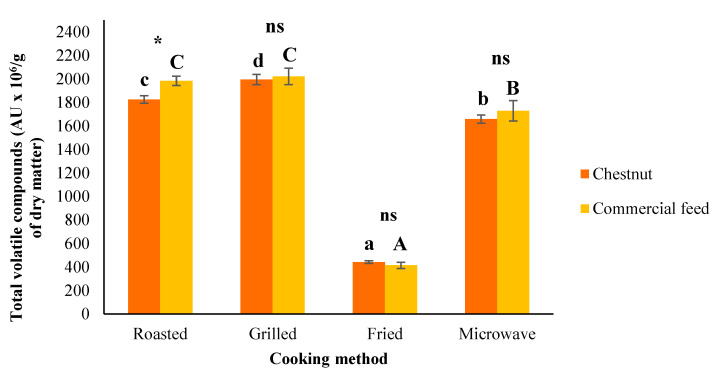
Effects of the inclusion of chestnut in the finishing diet and cooking methods on total volatile compounds of Celta pig *Biceps femoris* (mean ± standard error). Influenced by feeding: * (*P* < 0.05); ** (*P* < 0.01); *** (*P* < 0.001); ns: no significant difference. Different letters within the same feeding regimen (chestnut (^a,d^) or commercial feed (^A,C^)) indicate significant differences for cooking methods (*P* < 0.05).

For their part, the amount of total volatile compounds was only significantly (*P* < 0.05) affected by diet in roasted samples. Even so, as we can see in the Figure 2, the CF pigs obtained higher values for total volatile compounds (except for the fried samples). Furthermore, this parameter was significantly (*P* < 0.001) affected by the cooking method. More concretely, the total volatile compounds were affected in the same way by culinary treatment for both diets (Figure 2). Thus, the maximum value for the quantity of total volatile compounds was found in the grilled slices (1995.78 AU × 10^6^/g dry matter for CH versus 2021.63 AU × 10^6^/g dry matter for CF) followed by roasted (1825.15 and 1984.17 AU × 10^6^/g dry matter for CH and CF, respectively) and microwaved samples (1658.13 AU × 10^6^/g dry matter for CH and 1729.28 AU × 10^6^/g dry matter for CF). Meanwhile the lowest total volatile compounds was detected in fried treatment (441.58 AU × 10^6^/g dry matter for CH and 414.36 AU × 10^6^/g dry matter for CF) coinciding with the outcomes obtained by Lorenzo and Domínguez [14].

**Figure 3:**
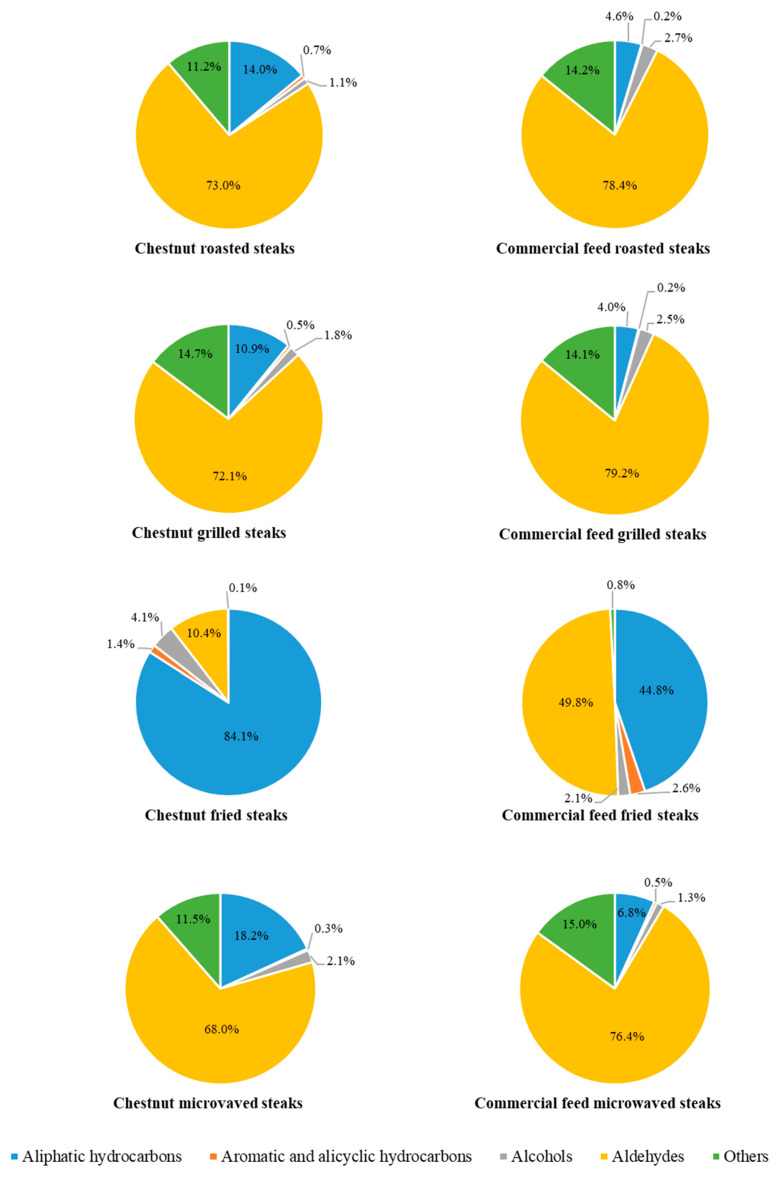
Volatile families of Celta pig *Biceps femoris* (expressed as percentages) affected by diet and by cooking method.

In relation to the family of aliphatic hydrocarbons, we have found values between 91.09 and 371.16 AU × 10^6^/g dry matter, which represent among 4.0 and 84.1% of the all volatile substances detected (Figure 3). Although the CH samples provided a significantly (*P* < 0.001) higher content in these compounds for all heat treatments, both diets showed the same trend. Thus, frying was the cooking method with the highest aliphatic hydrocarbon content, followed by microwaved, roasted and grilled. Additionally, after aldehydes, aliphatic hydrocarbons were the second most abundant volatile family (without taking into account the group of “others”, where five different compounds were arranged). Although there was an exception since in fried CH samples it was observed that the aliphatic hydrocarbons were the main group (Figure 3). In spite of its high presence, previous research has determined that this group is not involved in cooked meat’s overall aroma [74] because they have high flavour thresholds that minimal contributes to flavour [75]. On the contrary, the group of aromatic and alicyclic hydrocarbons, even showing low concentrations can have a large impact on aroma. This is because this group possesses some aromatic substances, such as pyrazines, that have a low odour threshold [73]. According to the above, low concentrations were obtained for these groups (percentages below 2.6%) in our work (Figure 3). Furthermore, the heat treatment that generated more substances of this type was frying for both diets. This could be due to the high temperature employed in this procedure (180 °C). Specifically, pyrazine, 3-ethyl-2,5-dimethyl- was detected in the case of fried chestnut samples, which obtained an average value of 5.68 AU × 10^6^/g dry matter. This compound could favour the global odour of these samples since pyrazines are related to savoury, nutty, roasted, and burnt odours [73]. On the other hand, alicyclic hydrocarbons do not contribute significantly to meat flavour because they have a high odour threshold [76].

Alcohols are mainly derived from the oxidative decomposition of lipids [73,75]. These substances are common components of cooked meat and meat products [75]. Nevertheless, they usually have higher odour thresholds and a low impact on the cooked meat flavour [76]. Even so, they occasionally contribute to desirable almond-like, toasted, woody and fatty-floral aroma notes [77,78]. In our case, alcohols were found in concentrations between 1.1 and 4.1% (Figure 3). Concretely, 1-pentanol was the alcohol that appeared in the highest concentration, being significantly (*P* < 0.05) higher for CF pigs, except for microwaved samples where this value was not significantly different (Table 4). Despite these differences, cooking also significantly (*P* < 0.001) affected both diets in a similar way. Like this, 1-pentanol was found to a greater extent in grilled CH samples (26.23 AU × 10^6^/g dry matter) and in roasted CF slices (47.65 AU × 10^6^/g dry matter). Meanwhile, frying showed the lowest values for the fried CF samples (8.51 AU × 10^6^/g dry matter), being even undetectable in CH slices. In addition to standing out for its high concentration compared to other alcohols, 1-pentanol is also important in the aroma of meat, since it has a low odour threshold. So this substance could benefit the global aroma owing to its mild, fruit and balsamic odour [10,74].

On the other hand, aldehydes were the most plentiful chemical family detected in cooked *Biceps femoris* slices affected by different heat treatments for both diets, with the exception of frying in CH diet, where aliphatic hydrocarbons were predominant (Figure 3). These volatile compounds are mainly generated from the oxidation of the unsaturated fatty acids [59], although they can also be originated through the Strecker degradation [79]. The aldehydes are likely the most important of the lipid-derived volatiles due to their low threshold odour, which contributes greatly to the flavour of meat [80]. In the case of cooked pork, aldehydes are mainly responsible for its aroma [80]. In this way, cooked grilled samples showed the highest values of total aldehyde content unlike fried samples (1439.59 vs. 45.84 AU × 10^6^/g dry matter for CH and 1601.21 vs. 206.17 AU × 10^6^/g dry matter for CF, respectively). In particular within aldehydes, hexanal was the major volatile compound observed in any cooking method according to other works [74,81], displaying values between 29.50 and 1412.07 AU × 10^6^/g dry matter (Table 4). Diet significantly (*P* < 0.05) affected hexanal quantity, apart from microwave cooking. Thus, CF seemed to increase the hexanal content in all cooking treatments. Even so, this compound showed the same trend in both diets, obtaining the maximum value for grilled samples, followed by roasted and microwaved muscle. Meanwhile, the lowest values were found for the fried samples. Then again, regarding cooking methods, the treatments used significantly (*P* < 0.001) affected the hexanal content as previously indicated. Nevertheless, these aftermaths do not agree with those showed by Broncano et al. [9] who determined that frying supplied the higher hexanal content in cooked pork. In the meantime, grilled loins showed the lowest hexanal value in their research. These differences could be motivated by an existence of inequalities in cooking techniques, such as temperatures, times and sample thickness. Like this, Broncano et al. [9] used, on grilled samples, higher temperatures (190 vs. 130–150 °C) and shorter times (4 vs. 10 min) than ours, which can run to less oxidation, so a lower hexanal content. In spite of that, other studies on foal meat [14,15] found results similar to ours, determining that frying led to lower hexanal values. This incident can be associated with the high content of α-tocopherol and polyphenols existing in olive oil, which could act to protect against oxidation [71]. In addition, hexanal is supposed to be one of the main indicators of lipid oxidation [79,81]. For this reason, a good correlation can be expected between TBARS value and this volatile compound. On this matter, we have found that TBARS value were strongly related to hexanal content in both diets, since a positive Pearson correlation was displayed (r = 0.798; *P* < 0.01, for chestnut feed pigs; and r = 0.831; *P* < 0.01, for commercial feed pigs). Heptanal was another important aldehyde in our research since it could undergo modifications as a consequence of diet. This is because heptanal is a degradation product of oleic acid [51,75] and the diets rich in this fatty acid (such as chestnut feed) can favour their accumulation in the intramuscular fat of pigs [6,18] and consequently the heptanal content in cooked samples. However, these differences were not found in the present study. A clear trend was not observed either, since the heptanal content was higher in grilled and fried CH samples, while, for the roasting and microwaving treatments, they were higher in CF samples.

Lastly, regarding the “others” group, the presence of one ketone should be pointed out, since these compounds are judged to have a high and peculiar influence on meat flavour [79]. The ketone found was the 2-heptanone, which was only significantly affected by diet in the case of frying treatment (*P* < 0.001). Furthermore, the different heat treatments significantly (*P* < 0.001) affected its content, and, in the same way, in both feeds (Table 4). Thus, the highest concentration was found for grilled samples (4.68 and 5.73 AU × 10^6^/g dry matter for CH and CF pig samples, respectively), while frying did not lead to their formation in CH slices, and, in the case of CF samples, this treatment generated the lowest quantity (1.12 AU × 10^6^/g dry matter). According to Mottram [73] and Domínguez et al. [15] it was found from Pearson test that 2-heptanone was also positively correlated with TBARS index (r = 0.747; *P* < 0.001 for CH samples; and r = 0.688; *P* < 0.001 for CF slices). To finish, the different presence of furan, 2-pentyl, is noted in accordance with the diet supplied. In that way, this substance was significantly (*P* < 0.01) higher in CH slices, apart from fried samples where this furan was not detected (Table 4). This incident could be justified by the inclusion of chestnut in the pig diet; because these fruits have a high concentration of linoleic acid [18], and this fatty acid can be decomposed by oxidation materializing furan, 2-pentil [75].

## 4. Conclusions

In general terms, the substitution of commercial feed for chestnut fruits in the finishing diet of the Celta pig modifies some physicochemical parameters of cooked *Biceps femoris* muscle. Thus, moisture content and cooking losses were slightly negatively affected by the inclusion of chestnut. Furthermore, the use of this fruit significantly increased both the intramuscular fat content and lipid oxidation. This could be due to the incident that chestnuts favour the fat deposition during the fattening, at the same time that the content of unsaturated fatty acids increases, which are more susceptible to oxidation. Regarding shear force, this parameter is not significantly affected by diet. Simultaneously, the employed chestnut also alters the amount of some volatile compounds, such as furan, 2-pentyl, which could be generated from the unsaturated fatty acids deposited throughout the chestnut.

On the other hand, the aftermaths achieved in this research displayed that the different thermal treatments (roasted, grilled, fried and microwaved) overall significantly affected the chemical composition, colour parameters, cooking loss, TBARS value and volatile profile of the *Biceps femoris* muscle, whereas the shear force was not significantly affected. Specifically, it should be pointed out that fried and microwaved treatments maintained the highest content of intramuscular fat. Moreover, frying also showed to be the one that generated the highest a* value, meanwhile the microwave method achieved the highest cooking losses. With respect to the lipid oxidation, frying was the technique that least affected this process because fried samples displayed the lowest TBARS value, and hexanal concentration. Besides, this thermal treatment showed the least concentration of volatile compounds, unlike grilled, which showed the highest content.

## Figures and Tables

**Table 1:** Effects of the inclusion of chestnut in the finishing diet and of cooking methods on chemical composition of Celta pig Biceps *femoris* muscle.

	Roasted	Grilled	Fried	Microwaved	SEM	T	FxT
**Moisture (%)**							
Chestnut	58.24 ^bc^	60.89 ^c^	54.58 ^ab^	53.41 ^a^	0.903	**	ns
Commercial feed	60.50	62.46	59.45	58.96	0.542	ns	
SEM	0.828	0.730	1.145	1.260			
F	ns	ns	*	*			
**Intramuscular Fat (%)**							
Chestnut	6.08	4.22	6.82	7.69	0.524	ns	ns
Commercial feed	2.67 ^a^	2.68 ^a^	4.07 ^b^	2.83 ^a^	0.904	**	
SEM	0.785	0.360	0.516	0.971			
F	*	*	**	**			
**Protein (%)**							
Chestnut	32.92 ^a^	32.06 ^a^	36.33 ^b^	36.07 ^b^	0.580	**	ns
Commercial feed	34.14 ^ab^	32.40 ^a^	34.00 ^ab^	35.99 ^b^	0.447	*	
SEM	0.548	0.566	0.752	0.528			
F	ns	ns	ns	ns			
**Ash (%)**							
Chestnut	1.66 ^a^	1.59 ^a^	1.79 ^b^	1.63 ^a^	0.026	*	ns
Commercial feed	1.83 ^c^	1.61 ^a^	1.75 ^bc^	1.70 ^ab^	0.026	*	
SEM	0.039	0.019	0.032	0.036			
F	*	ns	ns	ns			

SEM: Standard error of the mean. F: significantly different values as influenced by feeding; * (*P* < 0.05); ** (*P* < 0.01); *** (*P* < 0.001); ns: no significant difference. T: significantly different values as influenced by cooking method; * (*P* < 0.05); ** (*P* < 0.01); *** (*P* < 0.001); ns: no significant difference. FxT: interaction of feeding and cooking method; * (*P* < 0.05); ** (*P* < 0.01); *** (*P* < 0.001); ns: no significant difference. ^a–c^ Means within the same row not followed by the same letter differ significantly (influence of cooking method) (*P* < 0.05).

**Table 2:** Effects of the inclusion of chestnut in the finishing diet and of cooking methods on color parameters, water holding capacity and textural parameters of Celta pig *Biceps femoris* muscle.

	Roasted	Grilled	Fried	Microwaved	SEM	T	FxT
**Color parameters**							
Lightness (L*)							
Chestnut	49.97 ^b^	51.13 ^b^	34.89 ^a^	51.22 ^b^	1.663	***	ns
Commercial feed	49.25 ^b^	52.32 ^b^	40.96 ^a^	51.33 ^b^	1.125	***	
SEM	1.113	0.824	1.611	0.981			
F	ns	ns	ns	ns			
Redness (a*)							
Chestnut	7.15 ^b^	6.31 ^a,b^	9.34 ^c^	5.89 ^a^	0.333	***	ns
Commercial feed	7.52 ^a^	6.39 ^a^	10.32 ^b^	6.34 ^a^	0.404	***	
SEM	0.298	0.260	0.416	0.224			
F	ns	ns	ns	ns			
Yellowness (b*)							
Chestnut	18.95	17.54	15.81	16.87	0.460	ns	*
Commercial feed	18.63 ^b,c^	17.53 ^a,b^	19.63 ^c^	17.40 ^a^	0.263	**	
SEM	0.242	0.175	0.924	0.431			
F	ns	ns	*	ns			
**Water holding capacity**							
**Cooking loss (%)**							
Chestnut	39.34 ^b^	35.90 ^a^	42.93 ^c^	45.43 ^c^	0.911	***	ns
Commercial feed	35.02 ^a^	33.46 ^a^	37.55 ^a,b^	40.66 ^b^	0.973	*	
SEM	1.048	1.235	1.364	0.981			
F	*	ns	*	**			
**Textural parameters**							
**Shear force (N)**							
Chestnut	73.95	73.50	81.18	77.09	2.893	ns	ns
Commercial feed	86.92	71.43	80.90	77.57	3.672	ns	
SEM	5.816	3.892	5.892	2.044			
F	ns	ns	ns	ns			

SEM: Standard error of the mean. F: significantly different values as influenced by feeding: * (*P* < 0.05); ** (*P* < 0.01); *** (*P* < 0.001); ns: no significant difference. T: significantly different values as influenced by cooking method; * (*P* < 0.05); ** (*P* < 0.01); *** (*P* < 0.001); ns: no significant difference. FxT: interaction of feeding and cooking method; * (*P* < 0.05); ** (*P* < 0.01); *** (*P* < 0.001); ns: no significant difference. ^a–c^ Means within the same row not followed by the same letter differ significantly (influence of cooking method) (*P* < 0.05).

**Table 3:** Effects of the inclusion of chestnut in the finishing diet and of cooking methods on volatile compounds grouped by chemical families and on total volatile compounds (expressed as AU × 10^6^/g dry matter) of Celta pig *Biceps femoris* muscle.

	Roasted	Grilled	Fried	Microwaved	SEM	T	FxT
**Aliphatic hydrocarbons**							
Chestnut	256.42 ^a^	216.93 ^a^	371.16 ^c^	300.98 ^b^	15.932	***	ns
Commercial feed	91.09 ^a^	81.56 ^a^	185.56 ^c^	117.23 ^b^	10.632	***	
SEM	31.332	26.260	35.717	35.807			
F	***	***	***	***			
**Aromatic and alicyclic hydrocarbons**							
Chestnut	12.29 ^b^	10.87 ^b^	5.98 ^a^	4.35 ^a^	0.895	***	***
Commercial feed	3.47 ^a^	3.77 ^a^	10.78 ^c^	8.18 ^b^	0.824	***	
SEM	1.709	1.384	1.028	0.759			
F	***	***	**	***			
**Alcohols**							
Chestnut	19.70	35.12	18.16	35.45	3.282	ns	***
Commercial feed	53.05 ^b^	50.49 ^b^	8.51 ^a^	22.20 ^a^	5.374	***	
SEM	7.354	4.291	4.515	3.739			
F	**	ns	ns	ns			
**Aldehydes**							
Chestnut	1332.71 ^c^	1439.59 ^d^	45.84 ^a^	1127.46 ^b^	143.844	***	ns
Commercial feed	1554.76 ^c^	1601.21 ^c^	206.17 ^a^	1321.84 ^b^	147.954	***	
SEM	45.510	44.048	32.297	51.907			
F	**	ns	***	*			
**Others**							
Chestnut	204.04 ^b^	293.27 ^c^	0.44 ^a^	189.90 ^b^	28.085	***	**
Commercial feed	281.80 ^b^	284.61 ^b^	3.33 ^a^	259.83 ^b^	31.391	***	
SEM	16.486	13.688	0.550	17.218			
F	**	ns	***	*			
**Total volatile compounds**							
Chestnut	1825.15 ^c^	1995.78 ^d^	441.58 ^a^	1658.13 ^b^	158.561	***	ns
Commercial feed	1984.17 ^c^	2021.63 ^c^	414.36 ^a^	1729.28 ^b^	172.060	***	
SEM	38.422	38.508	13.812	45.688			
F	*	ns	ns	ns			

SEM: Standard error of the mean. F: significantly different values as influenced by feeding: * (*P* < 0.05); ** (*P* < 0.01); *** (*P* < 0.001); ns: no significant difference. T: significantly different values as influenced by cooking method; * (*P* < 0.05); ** (*P* < 0.01); *** (*P* < 0.001). FxT: interaction of feeding and cooking method; * (*P* < 0.05); ** (*P* < 0.01); *** (*P* < 0.001); ns: no significant difference. ^a–c^ Means within the same row not followed by the same letter differ significantly (influence of cooking method) (*P* < 0.05).

**Table 4:** Effects of the inclusion of chestnut in the finishing diet and of cooking methods on the volatile compounds (expressed as AU × 10^6^/g dry matter) of Celta pig *Biceps femoris* muscle.

	Roasted	Grilled	Fried	Microwaved	SEM	T	FxT
**Aliphatic hydrocarbons**							
**2,2,4,4-Tetramethyloctane**							
Chestnut	7.48	7.30	8.31	8.94	0.335	ns	ns
Commercial feed	5.06 ^b^	4.59 ^b^	3.84 ^a^	4.80 ^b^	0.156	*	
SEM	0.532	0.723	0.855	0.817			
F	**	*	***	***			
**3-Tridecene, (E)-**							
Chestnut	0.00 ^a^	0.00 ^a^	0.00 ^a^	3.01 ^b^	0.369	***	***
Commercial feed	0.00	0.00	0.00	0.00	0.000	ns	
SEM	0.000	0.000	0.000	0.649			
F	ns	ns	ns	**			
**Decane**							
Chestnut	18.9 ^b^	7.10 ^a^	26.75 ^c^	18.53 ^b^	1.930	***	***
Commercial feed	3.93	4.76	5.80	4.27	0.547	ns	
SEM	2.884	1.002	4.126	2.804			
F	***	ns	***	***			
**Decane, 2,3,5-trimethyl-**							
Chestnut	13.59 ^c^	9.48 ^b^	0.00 ^a^	13.33 ^c^	1.519	***	***
Commercial feed	0.00	0.00	0.00	0.00	0.000	ns	
SEM	2.588	2.041	0.000	2.557			
F	***	**	ns	***			
**Dodecane**							
Chestnut	32.54 ^b^	17.40 ^a^	32.02 ^b^	33.67 ^b^	1.909	***	ns
Commercial feed	13.18 ^b^	4.32 ^a^	16.15 ^b^	18.83 ^c^	1.596	***	
SEM	3.772	2.629	3.169	3.239			
F	***	***	***	**			
**Dodecane, 2,6,11-trimethyl-**							
Chestnut	0.00 ^a^	0.00 ^a^	0.00 ^a^	5.66 ^b^	0.675	***	***
Commercial feed	0.00	0.00	0.00	0.00	0.000	ns	
SEM	0.000	0.000	0.000	1.176			
F	ns	ns	ns	**			
**Dodecane, 3-methyl-**							
Chestnut	3.26 ^b^	0.00 ^a^	0.00 ^a^	6.51 ^c^	0.747	***	***
Commercial feed	0.00	0.00	0.00	0.00	0.000	ns	
SEM	0.723	0.000	0.000	1.296			
F	**	ns	ns	***			
**Heptane**							
Chestnut	2.17 ^b^	0.00 ^a^	0.00 ^a^	0.00 ^a^	0.250	***	***
Commercial feed	0.00 ^a^	0.00 ^a^	9.38 ^b^	0.00 ^a^	1.067	***	
SEM	0.429	0.000	1.820	0.000			
F	***	ns	***	ns			
**Heptane, 2,2,4,6,6-pentamethyl-**							
Chestnut	44.06 ^a^	40.42 ^a,b^	52.85 ^c^	47.25 ^b^	1.408	**	***
Commercial feed	34.44 ^b^	34.46 ^b^	31.00 ^a^	29.86 ^a^	0.621	**	
SEM	2.087	1.363	4.161	3.449			
F	**	*	***	***			
**Heptane, 2,2,4-trimethyl-**							
Chestnut	0.00 ^a^	0.00 ^a^	16.88 ^b^	0.00 ^a^	2.037	***	***
Commercial feed	0.00	0.00	0.00	0.00	0.000	ns	
SEM	0.000	0.000	3.564	0.000			
F	ns	ns	**	ns			
**Heptane, 3,3,5-trimethyl-**							
Chestnut	9.76 ^b^	0.00 ^a^	15.27 ^b^	0.00 ^a^	2.001	**	***
Commercial feed	0.00	0.00	0.00	0.00	0.000	ns	
SEM	2.006	0.000	3.549	0.000			
F	**	ns	*	ns			
**Heptane, 3-ethyl-**							
Chestnut	4.42 ^a^	4.19 ^a^	8.61^b^	8.51 ^b^	0.771	*	ns
Commercial feed	0.00 ^a^	1.84 ^b^	1.92 ^b^	2.45 ^b^	0.308	**	
SEM	0.888	0.733	1.447	1.343			
F	***	ns	**	**			
**Heptane, 3-methylene-**							
Chestnut	8.14 ^b^	2.79 ^a^	6.99 ^b^	6.62 ^b^	0.690	*	***
Commercial feed	0.00 ^a^	2.92 ^b^	0.00 ^a^	3.36 ^b^	0.481	**	
SEM	1.646	0.471	1.412	0.855			
F	***	ns	***	*			
**Hexane**							
Chestnut	11.36 ^a^	73.73 ^c^	50.92 ^b^	8.67 ^a^	7.155	***	***
Commercial feed	8.91 ^a^	8.31 ^a^	56.58 ^b^	11.69 ^a^	5.353	***	
SEM	1.273	12.495	1.721	1.799			
F	ns	***	ns	ns			
**Hexane, 2,2,4-trimethyl-**							
Chestnut	0.00 ^a^	0.00 ^a^	9.86 ^b^	0.00 ^a^	1.199	***	***
Commercial feed	0.00	0.00	0.00	0.00	0.000	ns	
SEM	0.000	0.000	2.105	0.000			
F	ns	ns	**	ns			
**Hexane, 2,2,5-trimethyl-**							
Chestnut	2.42 ^a^	2.53 ^a^	17.49 ^b^	10.55 ^a,b^	2.051	**	**
Commercial feed	1.06 ^b^	0.00 ^a^	1.92 ^c^	1.45 ^b,c^	0.211	***	
SEM	0.434	0.550	3.832	1.889			
F	ns	**	*	**			
**Hexane, 3,3-dimethyl-**							
Chestnut	7.40 ^b^	0.00 ^a^	0.00 ^a^	0.00 ^a^	0.896	***	***
Commercial feed	0.00	0.00	0.00	0.00	0.000	ns	
SEM	1.569	0.000	0.000	0.000			
F	**	ns	ns	ns			
**Nonadecane**							
Chestnut	1.50 ^b^	0.00 ^a^	2.73 ^c^	0.00 ^a^	0.314	***	***
Commercial feed	0.00	0.00	0.00	0.00	0.000	ns	
SEM	0.287	0.000	0.559	0.000			
F	***	ns	**	ns			
**Nonane, 3-methyl-**							
Chestnut	5.21 ^b^	3.15 ^a^	7.74 ^c^	5.12 ^b^	0.508	**	***
Commercial feed	0.00 ^a^	0.00 ^a^	0.00 ^a^	2.26 ^b^	0.262	***	
SEM	1.003	0.707	1.504	0.596			
F	***	***	***	**			
**Octane**							
Chestnut	7.88	5.80	8.18	5.91	0.542	ns	ns
Commercial feed	6.52	5.04	11.20	4.26	1.078	ns	
SEM	0.678	0.850	1.686	0.559			
F	ns	ns	ns	ns			
**Octane, 2,2-dimethyl-**							
Chestnut	0.00 ^a^	0.00 ^a^	0.00 ^a^	15.54 ^b^	1.904	***	***
Commercial feed	0.00 ^a^	0.00 ^a^	3.94 ^b^	0.00 ^a^	0.500	***	
SEM	0.000	0.000	0.892	3.350			
F	ns	ns	*	**			
**Octane, 3-methyl-6-methylene-**							
Chestnut	8.53 ^b^	0.00 ^a^	10.31 ^b^	0.00 ^a^	1.375	***	***
Commercial feed	0.00	0.00	0.00	0.00	0.000	ns	
SEM	1.842	0.000	2.157	0.000			
F	**	ns	**	ns			
**Pentane, 2,3,3-trimethyl-**							
Chestnut	9.21 ^a,b^	5.86 ^a^	13.83 ^b,c^	17.79 ^c^	1.435	***	ns
Commercial feed	0.00 ^a^	0.00 ^a^	9.89 ^b^	7.12 ^b^	1.318	***	
SEM	2.036	1.382	1.693	2.220			
F	**	*	ns	**			
**Pentane, 2,3,4-trimethyl-**							
Chestnut	2.89 ^a^	1.16 ^a^	8.04 ^b^	4.79 ^a,b^	0.860	**	*
Commercial feed	1.00	1.51	2.00	1.96	0.194	ns	
SEM	0.469	0.315	1.581	0.571			
F	*	ns	**	***			
**Pentane, 3-ethyl-2-methyl-**							
Chestnut	0.00 ^a^	0.00 ^a^	0.00 ^a^	12.48 ^b^	1.460	***	***
Commercial feed	0.00	0.00	0.00	0.00	0.000	ns	
SEM	0.000	0.000	0.000	2.520			
F	ns	ns	ns	***			
**Tridecane**							
Chestnut	8.67 ^b^	6.04 ^a^	6.98 ^a^	6.42 ^a^	0.334	**	***
Commercial feed	2.98 ^a^	2.47 ^a^	4.11 ^b^	4.88 ^b^	0.292	**	
SEM	1.109	0.717	0.605	0.424			
F	***	***	**	ns			
**Undecane**							
Chestnut	47.02 ^b^	19.38 ^a^	57.02 ^c^	50.92 ^b,c^	3.841	***	***
Commercial feed	11.02 ^a^	7.68 ^a^	23.99 ^b^	20.03 ^b^	2.141	**	
SEM	6.881	2.460	6.568	6.223			
F	***	**	***	***			
**Undecane, 3-methyl-**							
Chestnut	0.00 ^a^	8.05 ^b^	10.40 ^b^	10.80 ^b^	1.238	***	***
Commercial feed	2.99 ^b^	3.68 ^b^	3.87 ^b^	0.00 ^a^	0.506	**	
SEM	0.610	1.275	1.294	2.142			
F	***	ns	***	***			
**Undecane, 4,6-dimethyl-**							
Chestnut	0.00 ^a^	2.55 ^b^	0.00 ^a^	0.00 ^a^	0.327	***	***
Commercial feed	0.00	0.00	0.00	0.00	0.000	ns	
SEM	0.000	0.586	0.000	0.000			
F	ns	*	ns	ns			
**Aromatic and cyclic hydrocarbons**							
**.alpha.-Pinene**							
Chestnut	0.00 ^a^	0.00 ^a^	0.00 ^a^	4.03 ^b^	0.457	***	***
Commercial feed	0.00	0.00	0.00	0.00	0.000	ns	
SEM	0.000	0.000	0.000	0.778			
F	ns	ns	ns	***			
**1R-.alpha.-Pinene**							
Chestnut	0.00	0.00	0.00	0.00	0.000	ns	***
Commercial feed	0.00 ^a^	0.00 ^a^	5.24 ^c^	2.39 ^b^	0.561	***	
SEM	0.000	0.000	0.999	0.453			
F	ns	ns	***	***			
**Cyclopentane, nonyl-**							
Chestnut	4.69 ^c^	2.35 ^b^	0.00 ^a^	0.00 ^a^	0.527	***	***
Commercial feed	0.00	0.00	0.00	0.00	0.000	ns	
SEM	0.916	0.505	0.000	0.000			
F	***	**	ns	ns			
**Diethyl Phthalate**							
Chestnut	0.00 ^a^	0.00 ^a^	0.31 ^b^	0.32 ^b^	0.049	**	ns
Commercial feed	0.00 ^a^	0.00 ^a^	0.41 ^c^	0.24 ^b^	0.047	***	
SEM	0.000	0.000	0.053	0.043			
F	ns	ns	ns	ns			
**Oxime-, methoxy-phenyl-_**							
Chestnut	7.59 ^b^	8.52 ^b^	0.00 ^a^	0.00 ^a^	1.064	***	***
Commercial feed	3.47 ^a^	3.77 ^a,b^	5.12 ^b,c^	5.55 ^c^	0.314	*	
SEM	0.884	0.933	1.036	1.056			
F	**	***	***	***			
**Pyrazine, 3-ethyl-2,5-dimethyl-**							
Chestnut	0.00 ^a^	0.00 ^a^	5.68 ^b^	0.00 ^a^	0.648	***	***
Commercial feed	0.00	0.00	0.00	0.00	0.000	ns	
SEM	0.000	0.000	1.105	0.000			
F	ns	ns	***	ns			
**Alcohols**							
**1-Decanol, 2-ethyl-**							
Chestnut	0.00 ^a^	1.54 ^b^	0.00 ^a^	0.00 ^a^	0.182	***	***
Commercial feed	0.00	0.00	0.00	0.00	0.000	ns	
SEM	0.000	0.315	0.000	0.000			
F	ns	**	ns	ns			
**1-Hexanol**							
Chestnut	0.00 ^a^	7.36 ^b^	0.00 ^a^	0.00 ^a^	0.837	***	***
Commercial feed	5.39 ^b^	4.71 ^b^	0.00 ^a^	0.00 ^a^	0.741	***	
SEM	1.035	0.912	0.000	0.000			
F	***	ns	ns	ns			
**1-Octanol, 2-butyl-**							
Chestnut	0.00 ^a^	0.00 ^a^	0.00 ^a^	6.09 ^b^	0.789	***	***
Commercial feed	0.00	0.00	0.00	0.00	0.000	ns	
SEM	0.000	0.000	0.000	1.417			
F	ns	ns	ns	*			
**1-Pentanol**							
Chestnut	17.65 ^b^	26.23 ^c^	0.00 ^a^	16.68 ^b^	2.628	***	*
Commercial feed	47.65 ^b^	45.77 ^b^	8.51 ^a^	22.20 ^a^	4.771	***	
SEM	6.777	4.724	1.840	1.482			
F	**	*	**	ns			
**2,3-Butanediol**							
Chestnut	0.00 ^a^	0.00 ^a^	18.16 ^b^	12.68 ^a,b^	3.144	*	ns
Commercial feed	0.00	0.00	0.00	0.00	0.000	ns	
SEM	0.000	0.000	5.295	3.712			
F	ns	ns	ns	ns			
**2-Isopropyl-5-methyl-1-heptanol**							
Chestnut	2.05 ^b^	0.00 ^a^	0.00 ^a^	0.00 ^a^	0.247	***	***
Commercial feed	0.00	0.00	0.00	0.00	0.000	ns	
SEM	0.432	0.000	0.000	0.000			
F	**	ns	ns	ns			
**Aldehydes**							
**2-Hexenal, (E)-**							
Chestnut	1.41 ^b^	1.48 ^b^	0.00 ^a^	0.00 ^a^	0.195	***	***
Commercial feed	1.36 ^c^	1.47 ^c^	0.00 ^a^	0.95 ^b^	0.154	***	
SEM	0.088	0.088	0.000	0.191			
F	ns	ns	ns	***			
**Butanal, 3-methyl-**							
Chestnut	0.00 ^a^	0.00 ^a^	4.88 ^b^	0.00 ^a^	0.556	***	ns
Commercial feed	0.00 ^a^	0.00 ^a^	4.88 ^b^	0.00 ^a^	0.575	***	
SEM	0.000	0.000	0.433	0.000			
F	ns	ns	ns	ns			
**Heptanal**							
Chestnut	28.01 ^b^	42.20 ^c^	11.46 ^a^	24.73 ^b^	3.002	***	ns
Commercial feed	32.89 ^c^	36.09 ^c^	10.20 ^a^	24.81 ^b^	2.734	***	
SEM	1.886	1.981	0.516	1.548			
F	ns	ns	ns	ns			
**Hexanal**							
Chestnut	1178.88 ^c^	1268.93 ^c^	29.50 ^a^	1031.90 ^b^	128.940	***	ns
Commercial feed	1412.07 ^c^	1475.43 ^c^	159.00 ^a^	1203.04 ^b^	138.668	***	
SEM	47.538	49.643	27.288	46.649			
F	***	*	**	ns			
**Octanal**							
Chestnut	40.04 ^b^	42.33 ^b^	0.00 ^a^	0.00 ^a^	5.380	***	***
Commercial feed	31.52 ^b,c^	33.60 ^c^	23.03 ^a^	25.67 ^a,b^	1.418	**	
SEM	1.948	2.502	4.430	4.921			
F	*	ns	***	***			
**Pentanal**							
Chestnut	84.36 ^c^	84.64 ^c^	0.00 ^a^	70.82 ^b^	9.094	***	***
Commercial feed	76.92 ^d^	54.62 ^b^	9.06 ^a^	67.38 ^c^	6.846	***	
SEM	2.081	6.050	1.736	2.039			
F	ns	***	***	ns			
**Others**							
**2-Heptanone**							
Chestnut	5.04 ^c^	4.68 ^c^	0.00 ^a^	3.78 ^b^	0.529	***	ns
Commercial feed	5.59 ^c^	5.73 ^c^	1.12 ^a^	3.47 ^a^	0.521	***	
SEM	0.219	0.293	0.213	0.358			
F	ns	ns	***	ns			
**4-Hydroxymandelic acid, ethyl ester, di-TMS**							
Chestnut	0.00	0.00	0.00	0.00	0.000	ns	***
Commercial feed	0.00 ^a^	0.00 ^a^	0.00 ^a^	2.59 ^b^	0.292	***	
SEM	0.000	0.000	0.000	0.496			
F	ns	ns	ns	***			
**Caprolactam**							
Chestnut	0.00 ^a^	0.00 ^a^	0.44 ^b^	0.00 ^a^	0.049	***	***
Commercial feed	0.49 ^b^	0.00 ^a^	0.31 ^b^	0.00 ^a^	0.060	***	
SEM	0.107	0.000	0.027	0.000			
F	**	ns	**	ns			
**Furan, 2-pentyl**							
Chestnut	8.41 ^b^	7.98 ^b^	0.00 ^a^	8.91 ^b^	0.965	***	***
Commercial feed	6.98 ^c^	5.78 ^b^	1.91 ^a^	5.48 ^b^	0.513	***	
SEM	0.385	0.490	0.364	0.728			
F	ns	**	***	**			
**n-Caproic acid vinyl ester**							
Chestnut	190.59 ^b^	280.61 ^c^	0.00 ^a^	177.21 ^b^	26.834	***	**
Commercial feed	268.75 ^b^	273.10 ^b^	0.00 ^a^	248.29 ^b^	30.423	***	
SEM	16.577	13.704	0.000	17.178			
F	**	ns	ns	*			

SEM: Standard error of the mean. F: significantly different values as influenced by feeding; * (*P* < 0.05); ** (*P* < 0.01); *** (*P* < 0.001); ns: no significant difference. T: significantly different values as influenced by cooking method; * (*P* < 0.05); ** (*P* < 0.01); *** (*P* < 0.001); ns: no significant difference. FxT: interaction of feeding and cooking method; * (*P* < 0.05); ** (*P* < 0.01); *** (*P* < 0.001); ns: no significant difference. ^a–d^ Means within the same row not followed by the same letter differ significantly (influence of cooking method) (*P* < 0.05).
